# Targeting the Epidermal Growth Factor Receptor Pathway in Chemotherapy-Resistant Triple-Negative Breast Cancer: A Phase II Study

**DOI:** 10.1158/2767-9764.CRC-24-0255

**Published:** 2024-10-29

**Authors:** Clinton Yam, Miral Patel, Holly A. Hill, Ryan Sun, Roland L. Bassett, Elisabeth Kong, Senthil Damodaran, Kimberly B. Koenig, Sausan Abouharb, Sadia Saleem, Ajit K. Bisen, Rashmi K. Murthy, David L. Ramirez, Gaiane M. Rauch, Beatriz E. Adrada, Rosalind P. Candelaria, Xiaoping Wang, Elizabeth A. Mittendorf, Alastair M. Thompson, Jason B. White, Elizabeth E. Ravenberg, Alyson R. Clayborn, Qing-Qing Ding, Daniel J. Booser, Oluchi Oke, Abenaa M. Brewster, Gabriel N. Hortobagyi, Nuhad K. Ibrahim, Jennifer K. Litton, Vicente Valero, Banu K. Arun, Debu Tripathy, Jeffrey T. Chang, Ken Chen, Anil Korkut, Stacy L. Moulder, Lei Huo, Bora Lim, Naoto T. Ueno

**Affiliations:** 1 Department of Breast Medical Oncology, The University of Texas MD Anderson Cancer Center, Houston, Texas.; 2 Department of Breast Imaging, The University of Texas MD Anderson Cancer Center, Houston, Texas.; 3 Department of Bioinformatics and Computational Biology, The University of Texas MD Anderson Cancer Center, Houston, Texas.; 4 Division of Basic Sciences, Department of Biostatistics, The University of Texas MD Anderson Cancer Center, Houston, Texas.; 5 Cancer Biology and Therapeutic Program, University of Hawai’i Cancer Center, Honolulu, Hawaii.; 6 Division of Breast Surgery, Department of Surgery, Brigham and Women’s Hospital, Boston, Massachusetts.; 7 Breast Oncology Program, Dana-Farber/Brigham Cancer Center, Boston, Massachusetts.; 8 Harvard Medical School, Boston, Massachusetts.; 9 Division of Surgical Oncology, Section of Breast Surgery, Baylor College of Medicine, Houston, Texas.; 10 Department of Pathology, The University of Texas MD Anderson Cancer Center, Houston, Texas.; 11 Department of Integrative Biology and Pharmacology, McGovern Medical School, Houston, Texas.

## Abstract

**Purpose:**

Epidermal growth factor receptor (EGFR) pathway activation causes chemotherapy resistance, and inhibition of the EGFR pathway sensitizes triple-negative breast cancer (TNBC) cells to chemotherapy in preclinical models. Given the high prevalence of EGFR overexpression in TNBC, we conducted a single-arm phase II study of panitumumab (anti-EGFR monoclonal antibody), carboplatin, and paclitaxel as the second phase of neoadjuvant therapy (NAT) in patients with doxorubicin and cyclophosphamide (AC)–resistant TNBC (NCT02593175).

**Patients and Methods:**

Patients with early-stage, AC-resistant TNBC, defined as disease progression or ≤80% reduction in tumor volume after four cycles of AC, were eligible for this study and received panitumumab (2.5 mg/kg i.v., every week × 13), paclitaxel (80 mg/m^2^ i.v. every week × 12), and carboplatin (AUC = 4 i.v., every 3 weeks × 4) as the second phase of NAT. A two-stage Gehan-type design was used to detect an improvement in the pathological complete response (pCR)/residual cancer burden class I (RCB-I) rate from 5% to 20%. Whole-exome sequencing was performed on diagnostic tumor biospecimens, where available.

**Results:**

From November 3, 2016, through August 23, 2021, 43 patients with AC-resistant TNBC were enrolled. The combined pCR/RCB-I rate was 30.2%. The most common treatment-related adverse events were neutropenia (72%) and anemia (61%), with 7 (16%), 16 (37%), and 8 (19%) patients experiencing grade 4 neutropenia, grade 3 neutropenia, and grade 3 anemia, respectively. No new safety signals were observed.

**Conclusions:**

This study met its primary endpoint (pCR/RCB-I = 30.2% vs. 5% in historical controls), suggesting that panitumumab should be evaluated as a component of NAT in patients with chemotherapy-resistant TNBC in a larger, randomized clinical trial.

**Significance:**

The epidermal growth factor receptor (EGFR) pathway has been implicated as a driver of chemotherapy resistance in triple-negative breast cancer (TNBC). Here, we evaluate the combination of panitumumab, carboplatin, and paclitaxel as the second phase of neoadjuvant therapy (NAT) in patients with AC-resistant TNBC. This study met its primary efficacy endpoint, and molecular alterations in EGFR pathway genes did not seem to influence response to the study regimen.

## Introduction

In the neoadjuvant setting, pathological complete response (pCR) after neoadjuvant chemotherapy is associated with excellent long-term oncologic outcomes in triple-negative breast cancer (TNBC) including event-free and overall survival (1). However, approximately 40% to 50% of patients with TNBC have residual disease after receiving neoadjuvant chemotherapy, which is associated with a significantly higher risk of metastatic recurrence in the first 2 to 3 years (1–3). Although the addition of pembrolizumab to anthracycline/taxane-based chemotherapy is now considered the standard of care in the neoadjuvant setting for TNBC, the absolute improvement in the pCR rate conferred by the addition of pembrolizumab to chemotherapy is modest (2, 4). Specifically, although the first interim analysis of the KEYNOTE-522 trial (*n* = 602), in which pathological response was the primary endpoint, demonstrated a 13.6% improvement in the pCR rate with the addition of pembrolizumab to chemotherapy (2), subsequent data (*n* = 1,174) suggested a smaller absolute difference of 7.5% (3). Therefore, additional strategies are urgently needed to achieve better outcomes for patients with chemotherapy-resistant early-stage TNBC.

The epidermal growth factor receptor (EGFR) is a receptor tyrosine kinase and one of four members of the transmembrane EGFR family of receptors (5). Our group and others have shown that EGFR plays a key role in signal transduction pathways that drive the malignant phenotype (5–9), and suppression of EGFR signaling in breast cancer has been reported to control cancer growth by a few key mechanisms, including suppression of cancer stem cell populations (10–13), enhanced apoptosis via suppression of downstream MAPK/PI3K pathways (14–18), reversal of epithelial–mesenchymal transition (19), and converting an immunosuppressive tumor microenvironment to an immunoactive phenotype (20). Of note, EGFR overexpression is more prevalent in TNBC than other breast cancer subtypes and is associated with shorter survival (8, 9, 21). Interestingly, inhibition of EGFR signaling sensitizes TNBC cells to chemotherapeutic agents commonly used in TNBC, such as doxorubicin (22, 23). Given the significant degree of EGFR overexpression in TNBC, we hypothesized that targeting EGFR in chemotherapy-resistant TNBC would be an effective strategy to overcome resistance.

Panitumumab is a fully humanized IgG2 monoclonal antibody (mAb) that directly binds to EGFR and has demonstrated significant antitumor activity in preclinical models (24). In clinical trials, panitumumab has demonstrated promising signals of activity and acceptable safety profiles when combined with chemotherapy in patients with breast cancer, both in the neoadjuvant (25, 26) and metastatic settings (27, 28). Thus, we hypothesized that the addition of anti-EGFR therapy would sensitize chemotherapy-resistant TNBC and improve response rates in this setting.

Based on this rationale, we conducted a single-center, phase II study to determine the efficacy of targeting EGFR signaling with panitumumab combined with paclitaxel and carboplatin in patients with anthracycline-resistant, early-stage TNBC.

## Materials and Methods

### Eligibility

Patients who were 18 years of age or older were eligible for the study if they had histologically confirmed nonmetastatic TNBC, defined as breast cancers with <10% estrogen receptor and progesterone receptor staining of invasive tumor cells by immunohistochemistry (IHC) that did not have evidence of human epidermal growth factor receptor 2 (HER2) overexpression and/or amplification according to HER2 testing guidelines from the American Society of Clinical Oncology/College of American Pathologists (29, 30). Demonstration of anthracycline resistance, defined as disease progression or ≤80% reduction in the product of three orthogonal axes after four cycles of anthracycline-based neoadjuvant chemotherapy, was required. Patients were required to have an Eastern Cooperative Oncology Group (ECOG) performance status of 1 or better, as well as adequate organ and marrow function.

Patients were ineligible if they had any of the following: tumors measuring <1.0 cm after completing anthracycline-based neoadjuvant therapy (NAT); peripheral neuropathy of grade 1 or higher; and prior treatment with paclitaxel, carboplatin, and/or radiation to the primary breast cancer or axillary lymph nodes.

The protocol was reviewed and approved by The University of Texas MD Anderson Cancer Center Institutional Review Board, and all patients provided written informed consent (NCT02593175).

### Study design and treatment

The primary objective of this study was to evaluate the efficacy of the combination of panitumumab, paclitaxel, and carboplatin (PaCT) in patients with doxorubicin and cyclophosphamide (AC)–resistant, early-stage TNBC. Panitumumab was administered intravenously (i.v.) at a dose of 2.5 mg/kg every week for 13 weeks, with the first dose given 7 days prior to the first dose of paclitaxel and carboplatin for pharmacologic induction. Paclitaxel and carboplatin were administered at 80 mg/m^2^ i.v. once a week for 12 weeks and AUC = 4 i.v. every 3 weeks for 12 weeks (four cycles), respectively. After completion of systemic therapy, patients underwent definitive surgery. Postoperative radiation and adjuvant systemic therapy were administered as per institutional guidelines.

Complete medical histories, physical examinations, hematologic and metabolic profiles, relevant imaging studies, and toxicity assessments were performed before and during treatment. Patients remained in the study until radiologic evidence of disease progression, unacceptable toxicity, or withdrawal of consent.

### Safety monitoring and dose modification

Toxicity assessments were performed for all patients while in the study using the National Cancer Institute's Common Terminology Criteria for Adverse Events, version 4.0. Serious adverse events were captured from the time of the first protocol-specific intervention until 30 days after the last dose of the drug, unless the participant withdrew consent.

Patients experiencing grade 1 or 2 treatment-related adverse events (TRAE) attributed to panitumumab were managed with supportive measures. Patients experiencing grade 3 or higher TRAEs related to panitumumab were managed with dose interruptions and/or modifications. Two dose reductions of panitumumab were permitted prior to study treatment being discontinued because of toxicity. Dose interruptions and/or modifications of paclitaxel and carboplatin were managed at the discretion of the treating oncologist as per standard of care.

### Response assessment

All patients in this study were evaluated for clinical response by physical examination and/or imaging at the discretion of the treating oncologist. Upon early discontinuation or completion of study therapy, patients underwent surgical resection. The residual cancer burden (RCB) index (31) was determined, with pCR (RCB-0) defined as the absence of residual invasive cancer in the resected breast specimen and all sampled regional lymph nodes by histologic evaluation (ypT0/Tis ypN0). Patients experiencing disease progression during NAT to such an extent that precluded definitive surgical resection were classified as having RCB-III.

### Immunohistochemistry

Pretreatment tumor tissue obtained prior to initiation of AC was submitted for stromal tumor-infiltrating lymphocyte (sTIL) and immunohistochemistry (IHC) assessment of programmed death ligand 1 (PD-L1), androgen receptor (AR), and phosphatase tensin homolog (PTEN; refs. 32, 33) by dedicated breast pathologists (L. Huo and Q.-Q. Ding). Details of tissue preparation and IHC methods have been previously described (32, 33). High sTIL tumors were defined as tumors with sTIL infiltration of ≥20% (34), and PD-L1 positivity was defined as a combined positive score (CPS; ref. 35) of at least 1.

### Whole-exome sequencing

Genomic DNA was extracted from fresh frozen core needle biopsies of tumors obtained at the time of diagnosis and quantified using the PicoGreen dsDNA quantification assay (Invitrogen). DNA quality assessment, library preparation, exome capture, and sequencing were performed as previously described (32). Resulting whole-exome sequencing (WES) BCL files were converted into FASTQ format and demultiplexed using CASAVA 1.8.2. The FASTQs were aligned to hg19 with the Burrows–Wheeler Aligner; aligned reads were deduplicated using Picard (Broad Institute) and had base quality score realignment performed using GATK Spark (36).

We used Mutect2 (v4.4.0, Broad Institute) to call single-nucleotide variants (SNV) and small indels using default parameters, and the GATK (Broad Institute) somatic hg19/B37 panel of normals (36). Matched normal samples were also available for eight samples and were used to call variants for the corresponding tumor samples. Mutations were annotated using Funcotator (v4.4.0, Broad Institute; ref. 36) with several public genome databases for downstream filtering and analysis.

Somatic mutations were filtered using a tiered approach. First, SNVs and indels that were in intronic and intergenic regions were removed, as were variants found in the 3′ and 5′ untranslated regions and flanking regions. Silent variants and those attributed to pseudogenes, mitochondrial DNA, and unknown genes were also filtered out. We then removed variants with a tumor variant allele fraction of less than 5% and with a sequencing depth of less than 50. Variants that were found in the exome panel of normal were also removed.

SNVs and indels found in the gnomAD database (bioRxiv 2022.03.20.485034) with a population variant allele fraction greater than 0.10% were removed. Accordingly, variants found in dbSNP were removed unless they existed in one or more public somatic mutation databases (37–39). Variants annotated as “benign” or “likely benign” with ClinVar (40) were then removed. HLA gene variants were removed for the purposes of our analyses because of the highly polymorphic nature of the region. Variants found in FLAGS genes (41) that occur in an overabundance in public exome databases and long genes were also filtered from our mutation calls (245 variants).

Somatic mutations were visualized using MAFtools (42). Tumor mutational burden (TMB) was defined as the number of somatic mutations per sequenced megabase.

### Whole-transcriptome sequencing

RNA was extracted (Norgen Total RNA Purification Kit; Cat. 37500), treated with DNase I to eliminate genomic DNA residues, and purified (AMPure XP beads; Beckman Coulter Life Sciences). cDNA was prepared (Ovation RNA-Seq System V2; NuGEN) and up to 200 ng of cDNA was sheared (Covaris E220*evolution* Focused-ultrasonicator). Library preparation and hybridization were performed (Sciclone G3 NGSx Workstation; PerkinElmer, Inc.) using Agilent SureSelectXT Low Input Reagent Kit with indexes 1 to 96 and Agilent SureSelect Human All Exon v.4 probes. 500 to 1,000 ng of library were hybridized as single-sample reactions and sequenced on the Illumina NovaSeq 6000 platform for 2 × 150 paired end reads using Cycle Sequencing v3 reagents (Illumina). Data were preprocessed using the STAR aligner (43), HTSeq-count (44), RSEM (45), and FastQC. Vanderbilt TNBC signatures were calculated using the TNBCtype web server.

### Statistical methods

The initial planned enrollment for this study was 37 patients. A Gehan two-stage design was used, with 14 patients being enrolled in the first stage. During the first stage, if at least one patient had a favorable response, defined as a pCR or RCB-I, 23 more patients were to be enrolled for a total of 37 patients. This design was associated with a 49%, 23%, 10%, and 4% chance of terminating early if the true pCR/RCB-I rate was 0.05, 0.10. 0.15, and 0.20, respectively. The selection of pCR/RCB-I = 5% as the null hypothesis is based on data from the GeparTrio trial (46) and Aberdeen study (see Supplementary Methods for the detailed rationale; ref. 47). In response to emerging data suggesting that event-free survival (EFS) differed between patients experiencing a pCR and those experiencing an RCB-I (48), the protocol was modified on July 8, 2021, to increase the planned enrollment to 47 patients to provide greater precision in estimating the pCR rate to the study treatment.

Comparisons between patients experiencing pCR/RCB-I and those with RCB-II/RCB-III were made using Fisher exact tests. Relationships among pCR, RCB, mutations in EGFR pathway genes, and TMB were described with a one-way analysis of variance (ANOVA) after assessing the normality of residuals. The Kaplan–Meier method was used to estimate the distribution of EFS, metastasis-free survival (MFS), and overall survival (OS). Patients who remained alive and event-free (EFS), alive and metastasis-free (MFS), or alive (OS) were censored at their last follow-up date. Reported *P* values from hypothesis testing were considered statistically significant if less than 0.05.

### Data availability

The data generated in this study are not publicly available because of language in the informed consent document signed by participants on this study but are available upon reasonable request from the corresponding author.

## Results

### Patients

**Figure 1:**
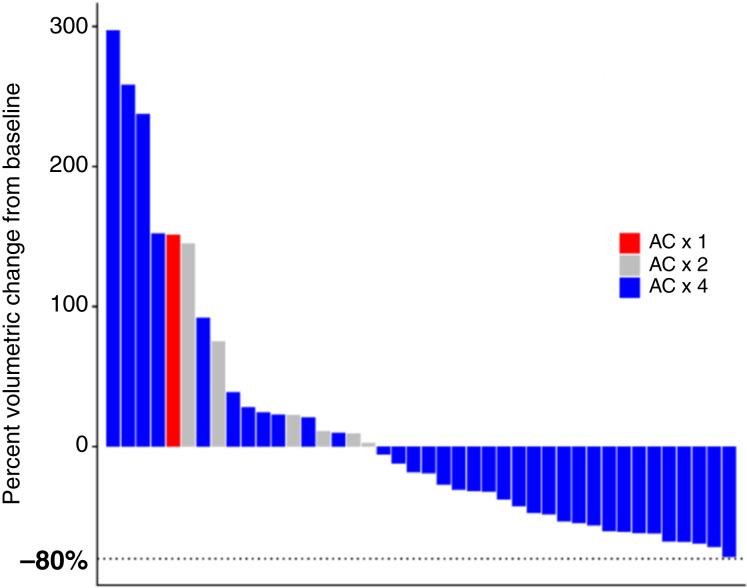
Response to AC. Waterfall plot showing percent sonographic volumetric change to initial AC chemotherapy received prior to enrollment in study. One of the 43 patients enrolled in this study is not represented on this plot as an ultrasound was not done at the end of AC. Colors represent the total number of cycles of AC received prior to enrollment in study (red = 1; gray = 2; blue = 4). Horizontal dashed line indicates an 80% decrease in calculated tumor volume when compared with baseline measurements.

Between November 3, 2016, and August 23, 2021, a total of 43 patients were enrolled and treated on this study. Baseline clinicopathologic characteristics are summarized in Table 1. The median age of diagnosis was 48.6 years [interquartile range (IQR), 42.9–56.1 years]. The majority of patients had clinically node-negative disease (58.1%) at the time of diagnosis. High sTIL infiltration (≥20%) and PD-L1 positivity (combined positive score ≥ 1) were found in tumors from 6 (14%) and 14 (33%) patients, respectively (Table 1). Thirty-five (81%) patients had tumors with <10% androgen receptor expression, and five (12%) patients had tumors with evidence of PTEN loss by IHC. All patients received neoadjuvant AC prior to enrollment in this study. Thirty-six (83.7%) patients received four cycles of AC, six patients (14.0%) received two cycles of AC, and one patient (2.3%) received one cycle of AC. The reason for early discontinuation of AC in all seven patients was an increase in tumor volume. Fig. 1 summarizes the volumetric change in tumor size to AC by ultrasound and the total number of cycles of AC received. One patient did not have an end of AC ultrasound performed, and disease progression on AC was determined based on clinical assessment in this patient. In addition to this patient, 18 other patients experienced an increase in tumor volume on AC prior to enrollment on this study (Fig. 1).

**Table 1:** Baseline clinicopathologic characteristics and associations with pathologic response

Characteristic	Total (*n* = 43)	pCR/RCB-I (*n* = 14)	RCB-II/RCB-III (*n* = 29)	*P* value
Median age at diagnosis (IQR), years	48.6 (42.9–56.1)	49.60 (45.1–61.7)	48.5 (42.3–54.6)	0.41
Race, *n* (%)				
White	25 (58)	6 (43)	19 (66)	0.20
Other	18 (42)	8 (57)	10 (34)	
Clinical T stage, *n* (%)				
T1/T2	30 (70)	12 (86)	18 (62)	0.16
T3/T4	13 (30)	2 (14)	11 (38)	
Clinical nodal status, *n* (%)				
Negative	25 (58)	10 (71)	15 (52)	0.33
Positive	18 (42)	4 (29)	14 (48)	
Histologic grade, *n* (%)				
2	5 (12)	0	5 (17)	0.16
3	38 (88)	14 (100)	24 (83)	
Ki67, *n* (%)				
≤35%	8 (19)	0	8 (28)	**0.043**
>35%	34 (79)	13 (93)	21 (72)	
Unknown	1 (2)	1 (7)	0	
Stromal TIL, *n* (%)				
<20%	34 (79)	12 (86)	22 (76)	1.00
≥20%	6 (14)	2 (14)	4 (14)	
Unknown	3 (7)	0	3 (10)	
PD-L1 (22C3) combined positive score, *n* (%)				
<1	26 (60)	7 (50)	19 (66)	0.18
≥1	14 (33)	7 (50)	7 (24)	
Unknown	3 (7)	0	3 (10)	
Androgen receptor expression, *n* (%)				
<10%	35 (81)	12 (86)	23 (79)	1.00
≥10%	5 (12)	2 (14)	3 (10)	
Unknown	3 (7)	0	3 (10)	
PTEN expression, *n* (%)				
Absent	5 (12)	2 (14)	3 (10)	1.00
Present	28 (65)	9 (64)	19 (66)	
Indeterminate/unknown	10 (23)	3 (21)	7 (24)	
Volumetric change to AC, *n* (%)				
Reduction (<0%)	24 (56)	7 (50)	17 (59)	0.53
Increase (>0%)	18 (42)	7 (50)	11 (38)	
Unknown	1 (2)	0	1 (3)	

### Efficacy

Among the 43 patients treated in this study, 13 patients (30.2%) experienced a pCR/RCB-I (pCR = 5 patients; RCB-I = 8 patients), 19 patients (44.2%) had RCB-II, and 10 patients (23.3%) had RCB-III, including 1 patient who did not undergo definitive surgery because of progression of disease.

Notably, patients with high Ki67 (>35%) tumors were more likely to experience a pCR/RCB-I as compared with patients with low/moderate Ki67 (≤35%) tumors (*P* = 0.043, Table 1). No other baseline clinicopathologic characteristics were significantly associated with pathological response, including sonographic response to AC (Table 1).

**Figure 2:**
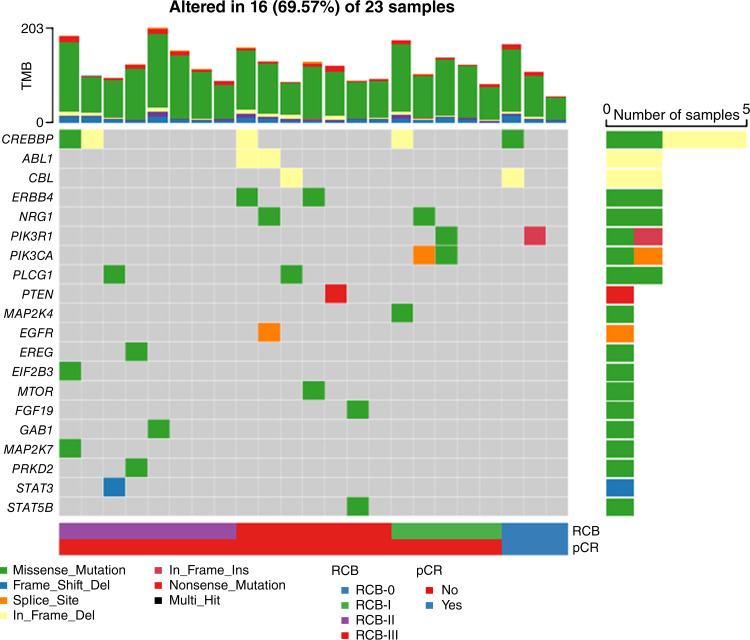
Genomic alterations and pathological response to therapy. Oncoplot showing somatic mutations in the EGFR pathway assessed by WES for each patient sample obtained at the time of diagnosis (*n* = 23). TMB was defined as the number of somatic mutations per megabase (Mb) of the sequenced genome.

Of the 43 patients enrolled in this study, 23 had sufficient archival tumor tissue obtained at the time of diagnosis for WES and RNA sequencing. The median number of somatic variants identified was 122 per sample (range, 67–203; Supplementary Fig. SF1). Somatic mutations in genes involved in the EGFR pathway (49) were found in tumors from 16 patients. Five patients had missense mutations in *CREBBP*, two other patients had mutations (one missense and one splice site) in *PIK3CA*, and one patient had a somatic nonsense mutation in *PTEN* identified by WES (Fig. 2). There were no significant differences in the frequency of EGFR pathway mutations between tumor samples from patients who experienced a pCR/RCB-I compared with those with higher residual disease burden (RCB-II/RCB-III). *TP53* mutations were detected in nine patients (39%), and gene mutations previously reported in TNBC were identified in our cohort (Supplementary Fig. SF2). Patients who experienced pCR/RCB-I seemed to have tumors with lower TMB compared with patients with RCB-II/RCB-III, but these differences were not statistically significant (Supplementary Fig. SF3). Of the 23 archival tumor samples available for RNA sequencing, one sample had to be excluded because of a low exonic rate, leaving 22 samples with transcriptomic data available for molecular subtyping. There were no significant associations between molecular subtypes of tumors from these 22 patients and pathological response (*P* = 0.743; Supplementary Table ST1).

**Figure 3:**
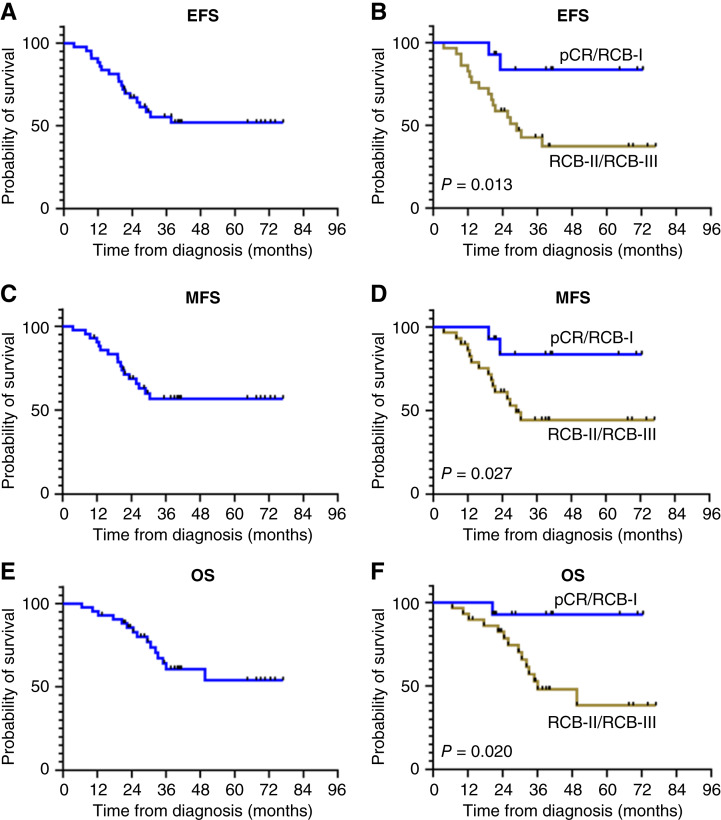
Survival outcomes. **A,** Kaplan–Meier plot of EFS for all patients. **B,** Kaplan–Meier plot of EFS separated by pathological response. **C,** Kaplan–Meier plot of MFS for all patients. **D,** Kaplan–Meier plot of MFS separated by pathological response. **E,** Kaplan–Meier plot of OS for all patients. **F,** Kaplan–Meier plot of OS separated by pathological response.

Thirty-seven patients (86%) received adjuvant radiation, and 23 patients (53%) received adjuvant systemic therapy (Supplementary Table ST2). At the time of data cutoff (March 25, 2023), 15 patients (34.9%) had died. The median duration of follow-up for patients last known to be alive was 39.5 months (range, 13.6–76.9 months). Kaplan–Meier estimates of EFS, MFS, and OS are shown in Fig. 3A–F. Compared with patients who were found to have RCB-II/RCB-III at the time of surgery, patients with pCR/RCB-I had improved EFS (median not reached vs. 28.9 months, *P* = 0.013; Fig. 3B), MFS (median not reached vs. 28.9 months, *P* = 0.027; Fig. 3D), and OS (median not reached vs. 36.2 months, *P* = 0.020; Fig. 3F).

### Toxicity

All 43 patients were evaluable for toxicity, having received at least one dose of panitumumab. TRAEs are summarized in Table 2. Seven patients (16.3%) experienced grade 4 neutropenia. The most common grade 3 TRAEs were neutropenia in 16 patients (37.2%), anemia in 8 patients (18.6%), leukopenia in 4 patients (9.3%), mucositis in 2 patients (4.7%), and thrombocytopenia in 2 patients (4.7%).

**Table 2:** TRAEs

TRAE		Grade (worst per patient, *N* = 43)			
	Total	Grade 4	Grade 3	Grade 2	Grade 1
Adverse event, *n* (%)					
Neutrophil count decreased	31 (72.1)	7 (16.3)	16 (37.2)	7 (16.3)	1 (2.3)
Anemia	26 (60.5)		8 (18.6)	15 (34.9)	3 (7)
White blood cell decreased	5 (11.6)		4 (9.3)	1 (2.3)	
Mucositis oral	27 (62.8)		2 (4.7)	6 (14)	19 (44.2)
Platelet count decreased	14 (32.6)		2 (4.7)	7 (16.3)	5 (11.6)
Rash maculopapular	20 (46.5)		1 (2.3)	2 (4.7)	17 (39.5)
Alanine aminotransferase increased	16 (37.2)		1 (2.3)	1 (2.3)	14 (32.6)
Hypomagnesemia	16 (37.2)		1 (2.3)	3 (7)	12 (27.9)
Hypokalemia	8 (18.6)		1 (2.3)	2 (4.7)	5 (11.6)
Dehydration	1 (2.3)		1 (2.3)		
Pharyngitis	1 (2.3)		1 (2.3)		
Syncope	1 (2.3)		1 (2.3)		
Urinary tract infection	1 (2.3)		1 (2.3)		
Dry skin	32 (74.4)			7 (16.3)	25 (58.1)
Fatigue	22 (51.2)			7 (16.3)	15 (34.9)
Peripheral sensory neuropathy	21 (48.8)			4 (9.3)	17 (39.5)
Pruritus	21 (48.8)			1 (2.3)	20 (46.5)
Rash acneiform	20 (46.5)			10 (23.3)	10 (23.3)
Diarrhea	18 (41.9)				18 (41.9)
Epistaxis	12 (27.9)				12 (27.9)
Aspartate aminotransferase increased	11 (25.6)			1 (2.3)	10 (23.3)
Nausea	11 (25.6)				11 (25.6)
Constipation	10 (23.3)				10 (23.3)
Dry mouth	5 (11.6)				5 (11.6)
Myalgia	5 (11.6)				5 (11.6)
Nail discoloration	5 (11.6)				5 (11.6)
Anorexia	4 (9.3)			1 (2.3)	3 (7)
Arthralgia	4 (9.3)				4 (9.3)
Dysgeusia	4 (9.3)			1 (2.3)	3 (7)
Erythema multiforme	4 (9.3)			1 (2.3)	3 (7)
Skin and subcutaneous tissue disorders–other	4 (9.3)			1 (2.3)	3 (7)
Dyspnea	3 (7)			2 (4.7)	1 (2.3)
Hot flashes	3 (7)			1 (2.3)	2 (4.7)
Sinus tachycardia	3 (7)			1 (2.3)	2 (4.7)
Vomiting	3 (7)				3 (7)
Breast pain	2 (4.7)			2 (4.7)	
Dizziness	2 (4.7)				2 (4.7)
Fever	2 (4.7)			1 (2.3)	1 (2.3)
Headache	2 (4.7)				2 (4.7)
Nail infection	2 (4.7)			1 (2.3)	1 (2.3)
Renal and urinary disorders–other	2 (4.7)			1 (2.3)	1 (2.3)
Skin ulceration	2 (4.7)			1 (2.3)	1 (2.3)
Vaginal dryness	2 (4.7)				2 (4.7)
Abdominal pain	1 (2.3)				1 (2.3)
Alkaline phosphatase increased	1 (2.3)			1 (2.3)	
Bone pain	1 (2.3)				1 (2.3)
Chills	1 (2.3)				1 (2.3)
Cough	1 (2.3)				1 (2.3)
Dry eye	1 (2.3)				1 (2.3)
Edema face	1 (2.3)			1 (2.3)	
Edema limbs	1 (2.3)			1 (2.3)	
Eye disorders–other	1 (2.3)				1 (2.3)
Fall	1 (2.3)			1 (2.3)	
Flushing	1 (2.3)				1 (2.3)
Gastroesophageal reflux disease	1 (2.3)				1 (2.3)
Hypernatremia	1 (2.3)				1 (2.3)
Hyponatremia	1 (2.3)				1 (2.3)
Hypotension	1 (2.3)				1 (2.3)
Nail ridging	1 (2.3)				1 (2.3)
Oral dysesthesia	1 (2.3)			1 (2.3)	
Oral pain	1 (2.3)				1 (2.3)
Pain	1 (2.3)				1 (2.3)
Palmar-plantar erythrodysesthesia syndrome	1 (2.3)				1 (2.3)
Papulopustular rash	1 (2.3)			1 (2.3)	
Reproductive system and breast disorders–other	1 (2.3)				1 (2.3)
Skin hyperpigmentation	1 (2.3)				1 (2.3)
Sore throat	1 (2.3)				1 (2.3)
Tinnitus	1 (2.3)				1 (2.3)
Watering eyes	1 (2.3)				1 (2.3)
Weight loss	1 (2.3)			1 (2.3)	

### Exposure to panitumumab

In this study, patients could receive up to a maximum of 13 doses of panitumumab, with one dose administered as monotherapy (dose −1) prior to initiation of 12 weeks of treatment with carboplatin plus paclitaxel (doses 1–12). Twenty-five patients (58.1%) received all 13 doses of panitumumab. Table 3 summarizes the panitumumab exposure of the remaining 18 (41.9%) patients along with reasons for dose reductions and/or discontinuation.

**Table 3:** Exposure to panitumumab

	Dose number													
Patient	−1	1	2	3	4	5	6	7	8	9	10	11	12	Reason for dose reduction/discontinuation
#2	2.5	2.5	2.5	0	2.5	2.5	2.5	0	0	0	0	0	0	Dose #3 held because of cytopenias; subsequent discontinuation due to disease progression
#3	2.5	2.5	2.5	2.5	2.5	0	0	2.5	2.5	0	0	0	0	Dose #5 and #6 held because of neutropenic sepsis; subsequent discontinuation due to persistent thrombocytopenia and neutropenia
#4	2.5	2.5	2.5	2.5	2.5	2.5	0	0	0	0	0	0	0	Discontinued because of neutropenia
#5	2.5	2.5	2.5	2.5	2.5	2.5	0	2.5	2.5	2.5	2.5	2.5	2.5	Dose #6 held because of neutropenia and thrombocytopenia
#6	2.5	2.5	2.5	2.5	2.5	2.5	2	0	2	2	2	2	2	Dose reduction at dose #6 due to mucositis; dose #7 held because of redness and eye nodule
#7	2.5	2.5	2.5	2.5	2.5	2.5	2.5	2.5	2.5	2.5	2	2	0	Dose reduction at dose #10 due to rash; subsequent discontinuation due to rash
#9	2.5	2.5	2.5	2.5	2.5	2.5	0	0	0	0	0	0	0	Discontinuation due to disease progression
#13	2.5	2.5	2.5	2.5	2.5	2.5	2.5	0	0	0	0	0	0	Discontinuation due to disease progression
#15	2.5	2.5	2.5	2.5	2.5	2.5	2.5	0	0	0	0	0	0	Discontinuation due to disease progression
#16	2.5	2.5	2.5	2.5	2.5	2.5	2.5	2.5	2.5	0	0	0	0	Discontinuation due to disease progression
#19	2.5	2.5	2.5	2.5	2.5	2.5	2.5	2.5	2.5	2.5	0	0	0	Discontinuation due to disease progression
#21	2.5	2.5	2.5	2.5	2.5	2.5	2.5	2.5	2.5	2.5	2.5	2.5	0	Dose #12 missed because of logistical reasons
#23	2.5	2.5	2.5	2.5	2.5	0	0	0	0	0	0	0	0	Discontinued because of rash
#28	2.5	2.5	2.5	2.5	2.5	2.5	2.5	2.5	2.5	2.5	2.5	0	2.5	Dose #11 held because of neutropenia and anemia
#30	2.5	2.5	2.5	2.5	2.5	2.5	2.5	2.5	2.5	0	2.5	2.5	2.5	Dose #9 held because of weakness and fatigue
#35	2.5	2.5	0	0	0	0	0	0	0	0	0	0	0	Discontinued because of rash
#42	2.5	2.5	2.5	2.5	2.5	2.5	2.5	2.5	2.5	2.5	0	0	0	Discontinuation due to disease progression
#43	2.5	0	0	0	0	0	0	0	0	0	0	0	0	Patient withdrew consent after dose −1 of panitumumab

## Discussion

We report the first study evaluating the use of panitumumab in combination with weekly paclitaxel and carboplatin in patients with early-stage, AC-resistant TNBC. The combination of panitumumab, carboplatin, and paclitaxel (PaCT) was well tolerated and demonstrated promising signals of clinical efficacy in this patient population.

In this study, we observed a pCR rate of 11.6% and a pCR/RCB-I rate of 30.2%. Although this is lower compared with the pCR rate observed in patients receiving neoadjuvant pembrolizumab plus chemotherapy (2), our study specifically selected for patients with chemotherapy-resistant TNBC including those who experienced increases in tumor volume while on AC, in which reported rates of pCR are as low as 2% to 5% (46, 47). Our data suggest that anti-EGFR therapy could augment responses to NAT in patients with chemotherapy-resistant TNBC.

The encouraging signal of activity observed in our study is in contrast to earlier studies of panitumumab added to carboplatin and paclitaxel or carboplatin and gemcitabine in TNBC that did not demonstrate improved response rates when compared with historical controls (27, 28). However, both of those studies were conducted in the metastatic setting, as opposed to the curative neoadjuvant setting. It is plausible that differences in tumor biology and/or drug exposure between metastatic and primary sites may account for the differing signals of efficacy. Given these observations, future studies evaluating panitumumab in TNBC may derive maximal impact by focusing on the neoadjuvant and treatment-resistant setting.

Our study has several limitations. First, the single-arm, nonrandomized design of our study precludes definitive conclusions on the role of panitumumab in this setting. However, to the best of our knowledge, this is the first study evaluating the role of anti-EGFR therapy in AC-resistant early-stage TNBC. Given the encouraging signal of activity, panitumumab should be explored further in future studies. Second, patients treated in our study did not receive neoadjuvant pembrolizumab, which was not approved in this setting at the time. Interestingly, a recent preclinical study showed that EGFR-targeted therapy switches the immunosuppressive tumor microenvironment to an immunoactive phenotype and improves the antitumor efficacy of anti–PD-(L)1 therapy (20). Given the similarity of the chemotherapy backbone used in our study and the KEYNOTE-522 trial, the impact of EGFR-targeted therapy on the tumor microenvironment, as well as the non-overlapping mechanisms of action and toxicity profiles of pembrolizumab and panitumumab, it would be interesting to determine if panitumumab augments the efficacy of the KEYNOTE-522 regimen in a future clinical trial. Third, our study did not have a mandatory tumor biopsy after completion of AC as standard of care and prior to initiation of study treatment, limiting our ability to draw conclusions about genotypic and phenotypic features of the tumor in the post-AC setting that may have influenced response to PaCT.

In conclusion, the combination of PaCT shows promise in the treatment of patients with AC-resistant early-stage TNBC. Further controlled studies evaluating the role of panitumumab are warranted in early-stage, treatment-resistant TNBC.

### Representativeness of study participants

Per AACR policy, information on representativeness of the study population is summarized in Supplementary Table ST3.

## Supplementary Material

SUPPLEMENTARY TABLE ST1 Molecular Subtypes and Pathological Response.

SUPPLEMENTARY TABLE ST2 Type of Surgery and Adjuvant Therapy Received.

SUPPLEMENTARY TABLE ST3 Representativeness of Study Participants.

Supplementary Figure SF1 Somatic mutations identified by whole exome sequencing. Oncoplot representing the most common somatic mutations assessed by whole exome sequencing (WES) in tumors obtained from patients (n = 23) at the time of diagnosis. Genes with mutations occurring in five or more patients are shown.

Supplementary Figure SF2 SUPPLEMENTARY FIGURE SF2. Somatic mutations in genes commonly altered in TNBC. Oncoplot showing somatic mutations reported as commonly prevalent in other TNBC cohorts.

Supplementary Figure SF3 SUPPLEMENTARY FIGURE SF3. Box plots showing relationship between tumor mutational burden and pathological response.
