# Supplementary material for: Targeting the Epidermal Growth Factor Receptor Pathway in Chemotherapy-Resistant Triple-Negative Breast Cancer: A Phase II Study
Source: Cancer Res Commun. 2024 Oct 29;4(10):2823–34. doi: 10.1158/2767-9764.CRC-24-0255 (PMC11520071; doi:10.1158/2767-9764.CRC-24-0255)
Supplement: SUPPLEMENTARY TABLE ST1 — Molecular Subtypes and Pathological Response. [file crc-24-0255_supplementary_table_st1_suppst1.docx]

**SUPPLEMENTARY TABLE ST1. Molecular Subtypes and Pathological Response.**

| **Molecular Subtype – n (%)** | **Total (n=22)** | **pCR/RCB-I (n=8)** | **RCB-II/RCB-III (n=14)** | **P value** |
| --- | --- | --- | --- | --- |
| Basal like 1 (BL1) | 1 | 0 | 1 (7) | 0.743 |
| Basal like 2 (BL2) | 3 | 1 (13) | 2 (14) |  |
| Immunomodulatory (IM) | 4 | 0 | 4 (29) |  |
| Luminal androgen receptor (LAR) | 2 | 1 (13) | 1 (7) |  |
| Mesenchymal (M) | 6 | 3 (38) | 3 (21) |  |
| Mesenchymal stem like (MSL) | 2 | 1 (13) | 1 (7) |  |
| Unstable (UNS) | 4 | 2 (25) | 2 (14) |  |
