# Supplementary material for: Targeting the Epidermal Growth Factor Receptor Pathway in Chemotherapy-Resistant Triple-Negative Breast Cancer: A Phase II Study
Source: Cancer Res Commun. 2024 Oct 29;4(10):2823–34. doi: 10.1158/2767-9764.CRC-24-0255 (PMC11520071; doi:10.1158/2767-9764.CRC-24-0255)
Supplement: SUPPLEMENTARY TABLE ST2 — Type of Surgery and Adjuvant Therapy Received. [file crc-24-0255_supplementary_table_st2_suppst2.docx]

**SUPPLEMENTARY TABLE ST2. Type of Surgery and Adjuvant Therapy Received.**

| **Patient** | **Tumor size at diagnosis (cm)** | **Clinical nodal status at diagnosis** | **Type of breast surgery** | **RCB status** | **Adjuvant radiation (Yes/No)** | **Adjuvant systemic therapy** | **Comments** |
| --- | --- | --- | --- | --- | --- | --- | --- |
| #1 | 3.5 | Negative | Mastectomy | RCB-II | No | No | Surgery was completed prior to publication of data from CREATE-X |
| #2 | 2.5 | Negative | Segmental mastectomy | RCB-II | Yes | Anastrozole | Surgery was completed prior to publication of data from CREATE-X; Patient had ER/PR-positive DCIS in the contralateral breast |
| #3 | 2.3 | Positive | Segmental mastectomy | pCR | Yes | No |  |
| #4 | 6.3 | Positive | Mastectomy | RCB-III | Yes | No | Patient decision |
| #5 | 2.3 | Negative | Segmental mastectomy | pCR | Yes | No |  |
| #6 | 3.3 | Positive | Mastectomy | RCB-III | Yes | Capecitabine |  |
| #7 | 6.8 | Positive | Mastectomy | RCB-II | Yes | No | Patient/Physician decision not to proceed with adjuvant systemic therapy |
| #8 | 3 | Negative | Segmental mastectomy | RCB-I | Yes | No | Patient/Physician decision not to proceed with adjuvant systemic therapy |
| #9 | 6.2 | Positive | N/A | RCB-III | No | No | Patient did not undergo definitive surgical resection |
| #10 | 2.5 | Negative | Segmental mastectomy | RCB-I | Yes | No | Patient/Physician decision not to proceed with adjuvant systemic therapy |
| #11 | 12 | Positive | Mastectomy | RCB-III | Yes | Capecitabine |  |
| #12 | 3.1 | Positive | Mastectomy | RCB-II | Yes | Capecitabine |  |
| #13 | 2.1 | Negative | Mastectomy | RCB-II | No | Capecitabine |  |
| #14 | 5.7 | Positive | Mastectomy | RCB-II | Yes | No | Patient/Physician decision not to proceed with adjuvant systemic therapy |
| #15 | 2.1 | Negative | Mastectomy | RCB-III | Yes | Capecitabine |  |
| #16 | 7.4 | Negative | Mastectomy | RCB-III | Yes | No | Found to have metastatic disease prior to initiation of adjuvant systemic therapy |
| #17 | 18.6 | Negative | Mastectomy | RCB-II | Yes | Capecitabine |  |
| #18 | 3.8 | Negative | Mastectomy | RCB-II | Yes | Capecitabine |  |
| #19 | 3.9 | Negative | Mastectomy | RCB-III | Yes | No | Found to have metastatic disease prior to initiation of adjuvant systemic therapy |
| #20 | 4.3 | Negative | Segmental mastectomy | RCB-II | Yes | Capecitabine |  |
| #21 | 2.4 | Positive | Segmental mastectomy | RCB-II | Yes | Capecitabine |  |
| #22 | 11.3 | Negative | Segmental mastectomy | RCB-II | Yes | No | Patient/Physician decision not to proceed with adjuvant systemic therapy |
| #23 | 1.7 | Negative | Mastectomy | pCR | No | No |  |
| #24 | 1.6 | Negative | Segmental mastectomy | RCB-I | Yes | Capecitabine |  |
| #25 | 2.4 | Positive | Segmental mastectomy | RCB-II | Yes | Anastrozole | Residual cancer was ER positive |
| #26 | 2.7 | Negative | Segmental mastectomy | RCB-I | Yes | No | Patient/Physician decision not to proceed with adjuvant systemic therapy |
| #27 | 3 | Negative | Mastectomy | RCB-II | No | No | Patient declined adjuvant systemic therapy |
| #28 | 4.9 | Positive | Segmental mastectomy | pCR | Yes | No |  |
| #29 | 2.2 | Negative | Segmental mastectomy | RCB-II | Yes | Capecitabine |  |
| #30 | 7 | Positive | Mastectomy | RCB-III | Yes | Capecitabine |  |
| #31 | 3.5 | Negative | Mastectomy | RCB-III | Yes | Capecitabine |  |
| #32 | 3.7 | Negative | Mastectomy | RCB-II | Yes | Capecitabine |  |
| #33 | 2.2 | Positive | Mastectomy | RCB-II | Yes | Trastuzumab-Pertuzumab | Residual cancer was HER2 positive |
| #34 | 1.6 | Positive | Mastectomy | RCB-I | Yes | No | Patient/Physician decision not to proceed with adjuvant systemic therapy |
| #35 | 4.4 | Negative | Segmental mastectomy | pCR | Yes | No |  |
| #36 | 2.3 | Negative | Segmental mastectomy | RCB-I | Yes | Capecitabine |  |
| #37 | 4.2 | Positive | Mastectomy | RCB-II | Yes | Capecitabine |  |
| #38 | 2.6 | Positive | Segmental mastectomy | RCB-II | Yes | Capecitabine |  |
| #39 | 1.8 | Positive | Segmental mastectomy | pCR | Yes | No |  |
| #40 | 2.4 | Positive | Segmental mastectomy | RCB-III | No | No | Found to have recurrent disease in the ipsilateral axilla prior to initiation of adjuvant systemic therapy |
| #41 | 8.5 | Negative | Mastectomy | RCB-I | Yes | Capecitabine |  |
| #42 | 6.6 | Negative | Mastectomy | RCB-I | Yes | Capecitabine |  |
| #43 | 2.4 | Negative | Mastectomy | RCB-II | Yes | Olaparib | Patient is a germline *BRCA1* mutation carrier |
