# Supplementary material for: Targeting the Epidermal Growth Factor Receptor Pathway in Chemotherapy-Resistant Triple-Negative Breast Cancer: A Phase II Study
Source: Cancer Res Commun. 2024 Oct 29;4(10):2823–34. doi: 10.1158/2767-9764.CRC-24-0255 (PMC11520071; doi:10.1158/2767-9764.CRC-24-0255)
Supplement: SUPPLEMENTARY TABLE ST3 — Representativeness of Study Participants. [file crc-24-0255_supplementary_table_st3_suppst3.docx]

**SUPPLEMENTARY TABLE ST3. Representativeness of Study Participants.**

| Cancare type(s)/subtype(s)/stage(s)/condition | Triple-negative breast cancer (TNBC) |
| --- | --- |
| Considerations related to: | |
| Sex | TNBC, as all other subtypes of breast cancer, is a predominantly female disease and is rare in men. Male breast cancer represents only between 0.5 and 1% of all breast cancers diagnosed each year. TNBC incidence is around 10-15% among all breast cancer subtypes in the overall largely female population, but its incidence in males is lower (4%) when compared to the other subtypes. |
| Age | The median age at the time of all breast cancer diagnoses is around 63, while in TNBC it is closer to 57. |
| Race/ethnicity | In the USA from 2014 to 2018, the overall breast cancer incidence rate was 127.1 cases per 100,000 among Black women and 132.5 cases per 100,000 among White women.  TNBC accounts for 27% and 16% of breast cancers in premenopausal Black and White women, respectively, and 15% and 9% breast cancers in postmenopausal Black and White women, respectively. |
| Geography | In the US, about 287,850 new cases of invasive breast cancer were diagnosed in 2021 and about 43,250 women die from breast cancer. |
| Other considerations | The breast cancer death rate among Black women surpassed that of White women in the mid-1980s and continued to increase until the mid-1990s after which it started to decline in parallel in both groups. However, the 5-year relative survival rate for all subtypes of breast cancer diagnosed during 2011 through 2017 was still lower, 82% among Black women versus 92% among White women. |
| Overall representativeness of this study | The age distribution of our study (median=48.6) is younger the average age distribution of TNBC in the literature.  In Texas, the Black population size is approximately 12% which is similar to the proportion of Black patients on our study at 19%.  As breast cancer is rare in men, no male patients with TNBC were recruited. |
