# Supplementary material for: Targeting the Epidermal Growth Factor Receptor Pathway in Chemotherapy-Resistant Triple-Negative Breast Cancer: A Phase II Study
Source: Cancer Res Commun. 2024 Oct 29;4(10):2823–34. doi: 10.1158/2767-9764.CRC-24-0255 (PMC11520071; doi:10.1158/2767-9764.CRC-24-0255)
Supplement: Supplementary Figure SF1 — Somatic mutations identified by whole exome sequencing. Oncoplot representing the most common somatic mutations assessed by whole exome sequencing (WES) in tumors obtained from patients (n = 23) at the time of diagnosis. Genes with mutations occurring in five or more patients are shown. [file crc-24-0255_supplementary_figure_sf1_suppsf1.docx]

**SUPPLEMENTARY FIGURE SF1**

**
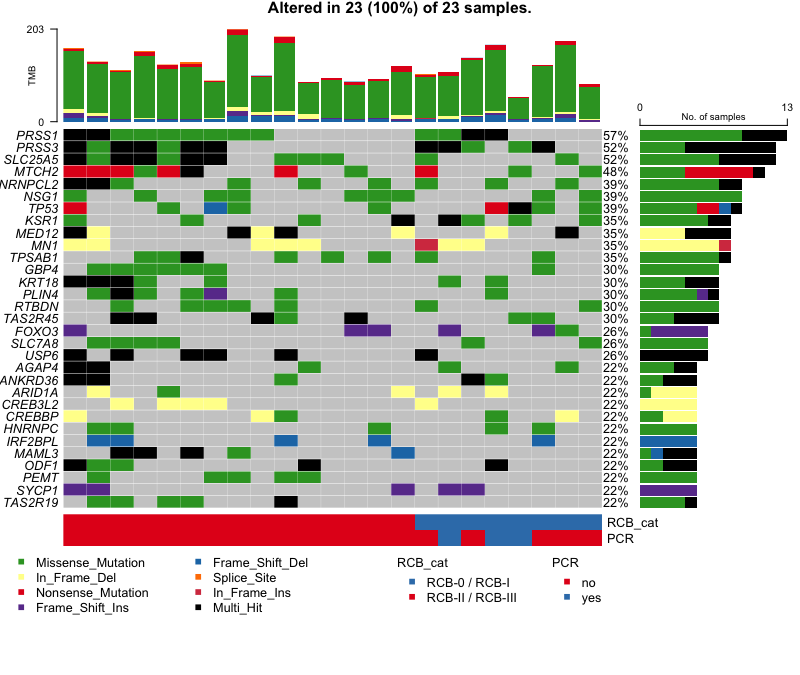
**

**SUPPLEMENTARY FIGURE SF1.**

**Somatic mutations identified by whole exome sequencing.** Oncoplot representing the most common somatic mutations assessed by whole exome sequencing (WES) in tumors obtained from patients (n=23) at the time of diagnosis. Genes with mutations occurring in five or more patients are shown.
