# Supplementary material for: Targeting the Epidermal Growth Factor Receptor Pathway in Chemotherapy-Resistant Triple-Negative Breast Cancer: A Phase II Study
Source: Cancer Res Commun. 2024 Oct 29;4(10):2823–34. doi: 10.1158/2767-9764.CRC-24-0255 (PMC11520071; doi:10.1158/2767-9764.CRC-24-0255)
Supplement: Supplementary Figure SF3 — Box plots showing relationship between tumor mutational burden and pathological response. [file crc-24-0255_supplementary_figure_sf3_suppsf3.docx]

**SUPPLEMENTARY FIGURE SF3**


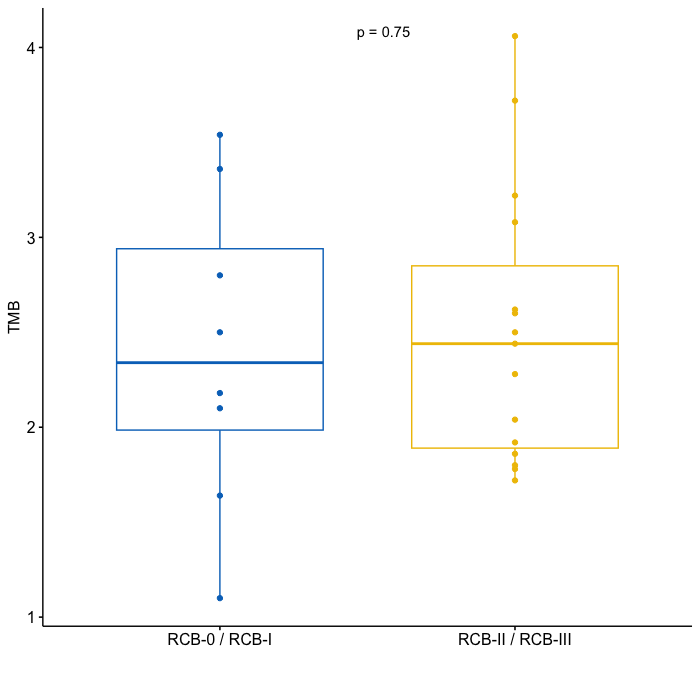


**SUPPLEMENTARY FIGURE SF3.**

Box plots showing relationship between tumor mutational burden and pathological response.
